# Distinct Biodistribution of Natural Killer Cell-Derived Exosomes in an Orthotopic A549 Lung Cancer Mouse Model: Implications for Potent Targeted Drug Delivery

**DOI:** 10.3390/life16040654

**Published:** 2026-04-13

**Authors:** Yen-Lien Chou, Kuo-Feng Hsu, Ssu-Han Chen, Shu-Yi Lin, Ming-Kung Yeh, Chi-Kang Lin, Yuan-Ming Tsai

**Affiliations:** 1 Division of Cardiology, Department of Internal Medicine, Tri-Service General Hospital, National Defense Medical University, Taipei 114202, Taiwan; archibaldkobe@gmail.com; 2 Division of Plastic and Reconstructive Surgery, Department of Surgery, Tri-Service General Hospital, National Defense Medical University, Taipei 114202, Taiwan; captain0416@gmail.com; 3 Department and Graduate Institute of Biochemistry, College of Biomedical Sciences, National Defense Medical University, Taipei 114201, Taiwan; chenssuhan@mail.ndmutsgh.edu.tw; 4 School of Pharmacy, College of Pharmacy, National Defense Medical University, Taipei 114201, Taiwan; shuyilin@mail.ndmutsgh.edu.tw (S.-Y.L.); mkyeh2004@precisionthera.com (M.-K.Y.); 5 Graduate Institute of Life Sciences, National Defense Medical University, Taipei 114201, Taiwan; 6 Department of Obstetrics and Gynecology, Tri-Service General Hospital, National Defense Medical University, Taipei 114202, Taiwan; 7 Division of Thoracic Surgery, Department of Surgery, Tri-Service General Hospital, National Defense Medical University, Taipei 114202, Taiwan; 8 Department & Graduate Institute of Medical Education & Bioethics, National Taiwan University College of Medicine, Taipei 100233, Taiwan

**Keywords:** lung cancer, orthotopic lung cancer mouse model, exosomes, NK-Exos, drug delivery system

## Abstract

**Background:** Exosomes (Exos) derived from immune cells are emerging as potent drug delivery vectors. However, their biodistribution in clinically relevant lung cancer models remains underexplored. This study aimed to evaluate the lung-homing ability of NK cell Exos (NK-Exos) compared to mesenchymal stem cell Exos (MSC-Exos) in an orthotopic lung cancer model. **Methods:** Male SCID mice were orthotopically injected with luciferase-tagged A549 cells into the left lung to establish the tumor model. Mice were randomized into four groups: G1 (Healthy Control), G2 (Tumor Control + PBS), G3 (Tumor + DiR-labeled NK-Exos; 5 µM DiR + 5–7 × 10^9^ Exo particles/100 μL/mouse), and G4 (Tumor + DiR-labeled MSC-Exos; 5 µM DiR + 5–7 × 10^9^ Exo particles/100 μL/mouse). Six hours (15 min, 1 h, 2 h, 4 h, 6 h) post-intravenous injection, ex vivo biodistribution was assessed using the MILabs Spectrum imaging system. **Results:** Umbilical cord blood-NK-Exos (UCB-NK-Exos; G3) exhibited superior accumulation in lung tissues compared to UCB-MSC-Exos (G4), suggesting enhanced pulmonary retention. Intra-pulmonary analysis revealed an asymmetric distribution, with significantly higher radiant efficiency in the right lung (non-tumor bearing) compared to the left lung (tumor injection site) across Exo-treated groups. **Conclusions:** UCB-NK-Exos demonstrate distinct lung-targeting properties superior to MSC-Exos, supporting their potential as therapeutic carriers.

## 1. Introduction

### 1.1. The Unmet Need in Lung Cancer Therapy

Lung cancer remains the leading cause of cancer-related mortality worldwide, with non-small-cell lung cancer (NSCLC) accounting for approximately 85% of all cases [1]. Despite significant advancements in targeted therapies and immunotherapies, prognosis remains poor for many patients. A primary obstacle to successful treatment is the presence of formidable physical and biological barriers within the tumor microenvironment (TME). Systemic administration of conventional chemotherapeutic agents and even modern tyrosine kinase inhibitors (TKIs) often suffer from poor intratumoral penetration and off-target toxicities [2]. Specifically, the high interstitial fluid pressure (IFP) and disorganized vasculature characteristic of NSCLC create a high-pressure gradient that impedes the convective transport of drugs into the tumor core [3]. Furthermore, the emergence of drug resistance, such as T790M or C797S mutations in EGFR-positive patients, underscores the urgent need for novel delivery systems capable of bypassing these barriers [4].

Conventional approaches (surgery, chemotherapy, radiotherapy) show limited efficacy in advanced or metastatic stages and often cause collateral damage to healthy tissues, while Natural Killer (NK) cell-based therapies, which show potential clinical application, are restricted by poor infiltration into solid tumors, short in vivo persistence, and complex manufacturing. In contrast, NK cell-derived exosomes (NK-Exos) retain cytotoxic activity and can more effectively penetrate the solid tumor microenvironment due to their nanometric size [5].

### 1.2. NK-Exos—Potential Drug Delivery Vehicle

Exosomes (Exos) are a specific subtype of extracellular vesicles (EVs) typically ranging from 30 to 150 nm in size and are characterized by the enrichment of conserved tetraspanins, including CD9, CD63, and CD81 [6]. Exos have gained significant attention as next-generation drug delivery systems due to their low immunogenicity and ability to cross biological barriers [7]. NK-Exos have emerged as a promising class of bio-missiles for targeted cancer therapy. Unlike mesenchymal stem cell-derived Exos (MSC-Exos), which are often regarded as neutral carriers with passive homing properties, NK-Exos inherit intrinsic cytotoxic mechanisms and natural tumor-homing capabilities from their parent cells. NK-Exos are enriched with activating receptors such as NKG2D, NKp30, and NKp46, allowing them to specifically recognize and bind to ligands overexpressed on lung cancer cells [8]. Additionally, their innate cargo, including perforin and granzymes, enables them to exert independent anti-tumor effects, potentially providing synergistic benefits when loaded with exogenous therapeutic agents [9]. However, most biodistribution studies have relied on subcutaneous xenograft models, which fail to replicate the organ-specific microenvironment of lung cancer. Orthotopic models, where tumor cells are implanted directly into the lungs, provide a more clinically relevant platform for evaluating therapeutic efficacy and biodistribution [10].

### 1.3. Study Aims

In this study, we utilized an orthotopic A549-luc lung cancer mouse model to compare the biodistribution of NK-Exos and MSC-Exos. We hypothesized that NK-Exos would exhibit distinct biodistribution patterns favorable for lung targeting, supporting their potential as efficient drug carriers.

## 2. Materials and Methods

### 2.1. Cell Culture and NK-Exo Isolation

Human lung adenocarcinoma cells (A549-luc) were cultured in DMEM supplemented with 10% FBS. Human umbilical cord (UC)-derived MSCs and human umbilical cord blood (UCB)-derived NK cells were isolated and expanded as previously described, with minor modifications. The collection of UC and UCB was approved by the Institutional Review Board, Tri-Service General Hospital, Taipei, Taiwan (IRB No. C202405130 and IRB No. B202405124, respectively). Briefly, UCB-MSCs were isolated via density gradient centrifugation and cultured in alpha-MEM, supplemented with 10% fetal bovine serum (FBS), following a protocol modified from a previous study [11]. For UCB-NK cells, CD56+ mononuclear cells were enriched and expanded in specialized growth medium supplemented with recombinant human IL-15 and IL-2 to promote proliferation and functional maturation, modified from the methods described by Spanholtz et al. [12]. Both cell types were maintained at 37 °C in a humidified atmosphere with 5% CO_2_.

Exos were isolated from the conditioned media of UC-MSCs and UCB-NK cells using the Exodus H600 system (Excellos, San Diego, CA, USA) modified from a previous method [13]. Briefly, for large-scale Exo production, MSCs at P4 were thawed and expanded to P5 in CellStack-10 (CF10) units. P5 cells were seeded into five CF10s at 3000 cells/cm^2^; upon reaching Day 3, the medium was replaced with serum-free and HPL-free media for a 48 h incubation before harvest. MSC tri-lineage differentiation tests were validated according to a previous study [14]. Simultaneously, NK cells were cultured until Day 27, followed by a medium exchange to initiate exosome production, with final collection on Day 30. A total of 9 L of conditioned media from both cell types were harvested and processed through the Exodus H600 system for high-purity Exos isolation, as previously described [13]. The cell and exosome preparation experiments were developed and conducted under the Good Tissue Practice (GTP) laboratory of the Precision Biotech Corp., Taiwan.

### 2.2. NK-Exos Analysis (Size, CD Markers)

The physical properties and protein profiles of the isolated NK-Exos were characterized to ensure purity and identity. The particle size distribution and concentration were determined using a NanoSight NS300 nanoparticle tracking analysis instrument (Malvern Panalytical, Malvern, UK). Samples were diluted with PBS to an optimal concentration, and three 60 s videos were captured and analyzed. For protein characterization, the presence of specific Exos markers and parent-cell markers was evaluated using an Amnis ImageStream Mk II imaging flow cytometer (Luminex Corporation, Austin, TX, USA). The following antibodies were used: PE-conjugated anti-human CD9 (clone HI9a; BioLegend, San Diego, CA, USA), APC-conjugated anti-human CD63 (clone H5C6; BioLegend, San Diego, CA, USA), and PerCP/Cy5.5-conjugated anti-human CD81 (clone 5A6; BioLegend, San Diego, CA, USA). High-resolution exosome images (50,000×) were obtained using a Hitachi S-3000N scanning electron microscope (Hitachi High-Technologies Corporation, Tokyo, Japan). 

### 2.3. Optimization of DiR Labeling Efficiency In Vitro

To establish high-sensitivity tracking of Exos in vivo, we optimized the fluorescent labeling conditions using the Spectrum system (MILabs U-OI, Houten, The Netherlands). The optimization process was designed to ensure robust signal detection while minimizing artifacts from unbound dye. Specifically, different quantities of MSC-Exos (ranging from 2 × 10^10^ to 2 × 10^11^ particles) were incubated with 5 µM DiR (1,1′-dioctadecyl-3,3,3′,3′-tetramethylindotricarbocyanine iodide; D12731, Invitrogen, LifeTechnologies, Carlsbad, CA, USA) for 1 h at 37 °C in the dark. To verify labeling specificity and remove free-dye residues, the mixture was purified using Amicon^®^ Ultra Centrifugal Filter, (MW 10 kDa, Merck, Darmstadt, Germany; UFC501096) and centrifuged at 14,000× *g* for 15 min at 4 °C, followed by a reverse spin at 14,000× *g* for 5 min at 4 °C. PBS was used as solution control. Imaging was performed with the following system parameters: exposure time: 20 s, iris setting: f/4.0, preamp: 1×, binning: 2 × 2, excitation wavelength: 710 nm, emission wavelength: 775 nm. The pseudocolor scale bar represents the radiant efficiency in photons per second (P/S).

### 2.4. Orthotopic Lung Cancer Model Establishment

All animal procedures were approved by the Institutional Animal Care and Use Committee (IACUC Protocol No: 2025-R403-032). Male SCID mice were anesthetized, and the 6th culture generation of A549-luc cells (3 × 10^6^ cells in PBS/Matrigel) were injected directly into the left lung parenchyma via the intercostal space (orthotopic surgery). The orthotopic lung cancer model was established following previously published protocols [15]. Tumor growth was monitored weekly via bioluminescence imaging.

### 2.5. Study Design and Animal Grouping

All animal experiments were conducted in accordance with the institutional guidelines (IACUC Protocol No. 2025-R403-032). Male SCID mice (NOD. Cg-Prkdc^scid^/JNarl) were used to establish the orthotopic lung cancer model. Following tumor verification via bioluminescence imaging, mice were randomized into four experimental groups as detailed below: Group 1 (Healthy Control, *n* = 1), healthy mice receiving PBS injection; Group 2 (Tumor Control, *n* = 3), A549-luc orthotopic tumor-bearing mice receiving PBS injection; Group 3 (UCB-NK-Exos, *n* = 4; 5 µM DiR + 5–7 × 10^9^ Exo particles), A549-luc orthotopic tumor-bearing mice receiving DiR-labeled UCB-NK-Exos; Group 4 (UC-MSC-Exos, *n* = 5; 5 µM DiR + 5–7 × 10^9^ Exo particles /100 μL/mouse), A549-luc orthotopic tumor-bearing mice receiving DiR-labeled UC-MSC-Exos.

### 2.6. MILabs Imaging and Biodistribution Analysis

Six hours post-injection (15 min, 1 h, 2 h, 4 h, 6 h), mice were euthanized. Major organs (lung, heart, liver, spleen, kidney, brain, intestine) were harvested. The fluorescence intensity of the Exos was quantified using the MILab Spectrum system (MILabs U-OI, Houten, The Netherlands). To analyze intra-pulmonary distribution, the left (tumor-bearing) and right lungs were separated and imaged. Data were analyzed using the instrument-provided MILabs U-OI software (OI-PP module, version 2.4.7; MILabs, Houten, The Netherlands) and expressed as average radiant efficiency ([p/s/cm^2^/sr]/[µW/cm^2^]). Imaging was performed with the following system parameters: exposure time: 60 s, iris setting: f/4.0, preamp: 1×, binning: 2 × 2, excitation wavelength: 710 nm, emission wavelength: 775 nm. The pseudocolor scale bar represents the radiant efficiency in photons per second (P/S).

### 2.7. Protein Profiling of NK-Exos

To characterize the protein cargo and surface signaling molecules of the isolated vesicles, a comprehensive protein profiling was performed using the RayBio^®^ Human Cytokine Antibody Array (C-Series) (RayBiotech Life, Inc., Peachtree Corners, GA, USA). Briefly, NK-Exos (2 × 10^11^ particles) were lysed using a specialized lysis buffer supplemented with a protease inhibitor cocktail to release the intra-vesicular and membrane-bound proteins. The array membranes, pre-coated with capture antibodies, were blocked with a blocking buffer for 30 min and subsequently incubated with the NK-Exos lysates at 4 °C overnight. After rigorous washing steps to remove non-specific binding, the membranes were incubated with a biotinylated antibody cocktail, followed by HRP-conjugated streptavidin. The protein signals were visualized using enhanced chemiluminescence (ECL) and captured by a high-resolution ChemiDoc imaging system controlled by Image Lab software version 6.1 (Bio-Rad Laboratories, Hercules, CA, USA). Signal intensities were quantified using ImageJ software version 1.54 (National Institutes of Health, Bethesda, MD, USA), with normalization against the internal positive and negative controls provided by the kit.

### 2.8. Statistical Analysis

Statistical analyses were performed using GraphPad Prism software (version 9.0; GraphPad Software, San Diego, CA, USA). All quantitative data are presented as mean ± standard error of the mean (SEM). Homogeneity of variance was assessed using the Brown–Forsythe test. A *p*-value > 0.05 indicated that the assumption of equal variance was satisfied. For comparisons of Exos biodistribution across multiple experimental groups, an ordinary one-way analysis of variance (ANOVA) was employed, followed by Tukey’s post hoc test for multiple pairwise comparisons. Statistical significance was defined at multiple levels and denoted by specific symbols in the figures: the hash symbol (#) represents a comparison between the PBS control group and the Exos-treated groups. The asterisk (*) represents a direct comparison between the UCB-NK-Exos and UCB-MSC-Exos groups. The level of significance is indicated as follows: ## or ** for *p* < 0.01; ### or *** for *p* < 0.001; and #### or **** for *p* < 0.0001. A *p*-value < 0.05 was considered statistically significant for all tests.

## 3. Results

### 3.1. Comprehensive Characterization of NK-Exos and MSC-Exos (Figure 1)

**Figure 1:**
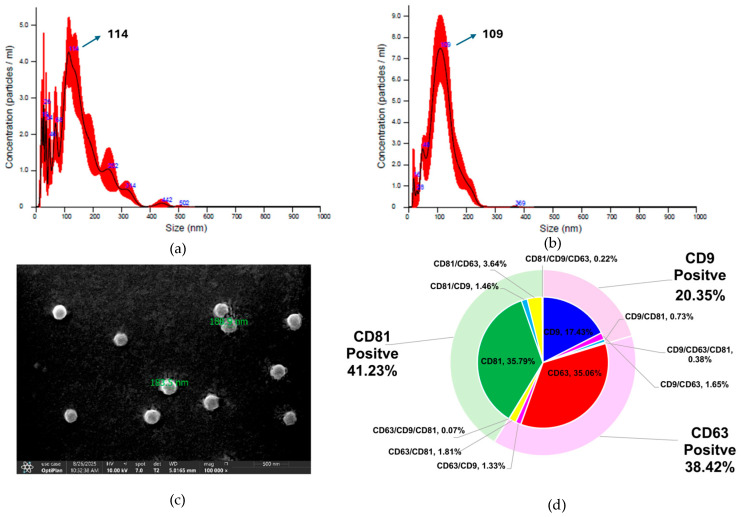
Characterization of UCB-derived MSC-Exos and NK-Exos. (**a**,**b**) Particle size distribution and concentration analysis. Representative nanoparticle tracking analysis profiles showing the size distribution of (**a**) MSC-Exos and (**b**) NK-Exos. (**c**) Morphological observation. Scanning electron microscopy image of NK-Exos at 50,000× magnification. (**d**) Surface marker profiling via flow cytometry. Quantitative analysis of classic extracellular vesicle markers on NK-Exos. (**a**) MSC-Exos: particle size analysis; (**b**) NK-Exos: particle size analysis; (**c**) NK-Exos particles under scanning electron microscope; (**d**) NK-Exos: CD marker analysis.

To verify the identity and purity of the isolated NK-Exos and MSC-Exos, we first analyzed their biophysical and biochemical characteristics. The mean diameter of the MSC-Exos particles was approximately 114 nm, which falls within the typical range for small Exos (Figure 1a), while the mean diameter of the NK-Exos particles was approximately 109 nm, which falls within the typical range for small Exos (Figure 1b). The scanning electron microscope results (under ×100,000) showed that the NK-Exos exhibited a characteristic round-shaped morphology (Figure 1c). To further quantify the molecular composition of the isolated vesicles, flow cytometric analysis was performed to determine the percentage distribution of key surface markers (Figure 1d). The majority of the isolated particles were confirmed as Exos, with CD9+, CD63+ and CD81+ populations accounting for 20.34%, 38.42% and 41.23% of the total count, respectively (Figure 1d).

### 3.2. Optimization of DiR Labeling Conditions for Exos Tracking (Figure 2)

**Figure 2:**
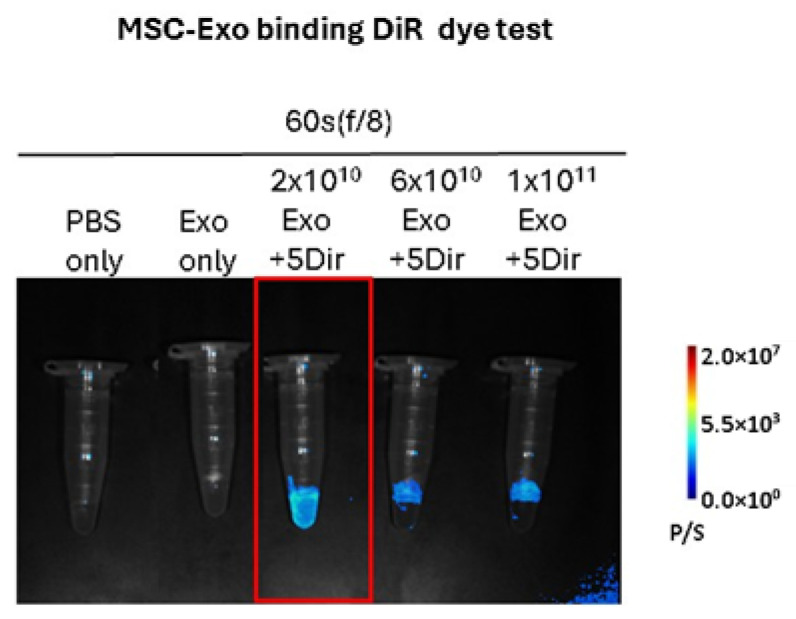
In vitro validation of DiR dye labeling efficiency on Exos. Representative fluorescence images of DiR-labeled Exos captured using the MILabs optical imaging system. The experiment included control groups (PBS only, and unlabeled Exo only) and experimental groups with increasing concentrations of Exos (2 × 10^10^, 6 × 10^10^, and 1 × 10^11^ particles) labeled with a fixed amount of DiR dye. The red box highlights the optimal labeling condition (2 × 10^10^ Exo + 5 µL DiR) used for the follow-up animal experiments.

To ensure accurate tracking of Exos in vivo, we first established the optimal labeling conditions using UCB-MSC-Exos (Figure S1). In vitro IVIS imaging revealed a dose-dependent increase in fluorescence intensity correlating with both Exo particle number and DiR concentration (Figure 2). Comparison between free DiR and DiR-labeled Exos after column purification demonstrated that the washing process effectively removed unbound dye, as evidenced by the minimal background signal in the free dye control group, while 5 µM DiR with 2 × 10^10^ Exo particles provided a robust signal-to-noise ratio sufficient for detection without potential dye aggregation or fluorescence quenching issues. Consequently, a concentration of 5 µM DiR with 2 × 10^10^ Exo particles was selected as the standardized condition for the subsequent biodistribution study in tumor-bearing mice.

### 3.3. Spatiotemporal Biodistribution of Exos in Orthotopic Lung Cancer Mice (Figure 3)

**Figure 3:**
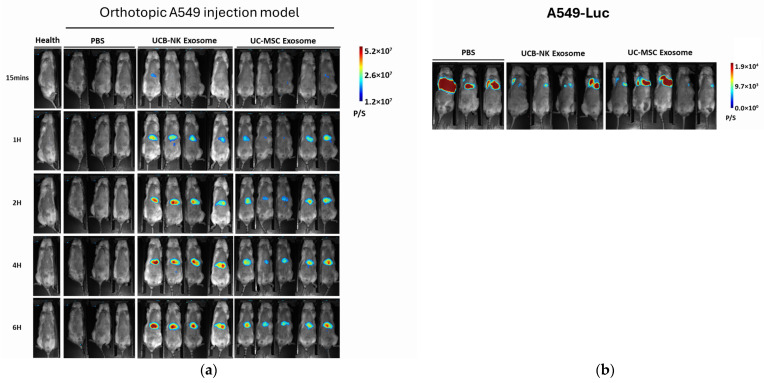
Spatiotemporal in vivo tracking of DiR-labeled Exos in an orthotopic lung cancer model. (**a**) Real-time biodistribution of UCB-derived MSC-Exos and NK-Exos. Serial whole-body fluorescence imaging was performed on mice following intrathoracic injection of A549-Luc cells to monitor the systemic fate of DiR-labeled exosomes. Images were captured at multiple time points (15 min, 1 h, 2 h, 4 h, and 6 h) post-intravenous administration. (**b**) Bioluminescence imaging of orthotopic A549-Luc tumors. To confirm the successful establishment and localization of the lung tumor prior to Exo administration, bioluminescence imaging was conducted. The persistent luciferase signal in the thoracic region across PBS, UCB-NK-Exos, and UC-MSC-Exos groups validates the site-specific growth of A549-Luc cells in the orthotopic model. The color scale represents bioluminescent intensity in photons per second (P/S). (**a**) Exosome tracking (DiR labeled); (**b**) A549-Luc.

Prior to the biodistribution analysis, the successful establishment and anatomical localization of the orthotopic lung cancer were verified via bioluminescence imaging in Groups 2, 3, and 4 (Figure 3b). To track the real-time systemic distribution and kinetics of the administered vesicles, we performed longitudinal bioluminescence and fluorescence imaging using the MILab Spectrum system. Following intravenous administration, both UCB-NK-Exos (G3) and UCB-MSC-Exos (G4) showed rapid systemic dissemination. At the earliest time point (15 min), fluorescence signals were primarily detected in the highly vascularized regions. Over the 6 h observation period, a time-dependent accumulation was observed in specific clearance organs. Consistent with the typical metabolic pathway of exogenous nanoparticles, the DiR-labeled Exos predominantly localized to the liver and spleen [16], reflecting sequestration by the mononuclear phagocyte system (Figure 3a).

### 3.4. Ex Vivo Biodistribution and Quantitative Analysis of Major Organs (Figure 4)

**Figure 4:**
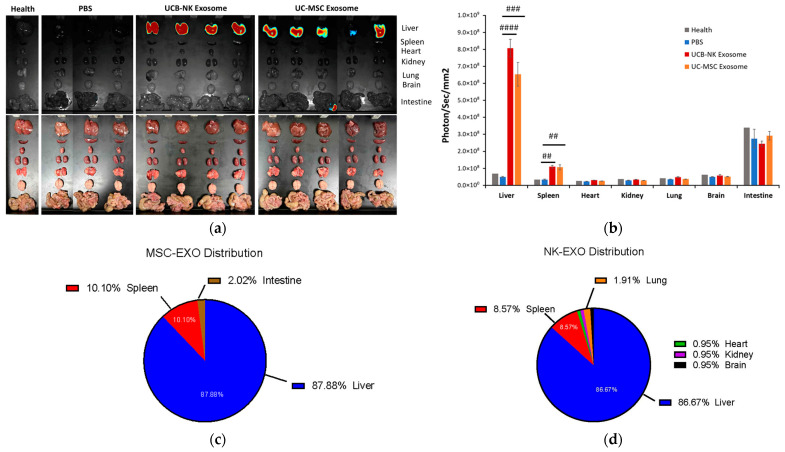
Quantitative biodistribution and organ-specific accumulation of Exos at 6 h post-injection. (**a**) Ex vivo fluorescence imaging of major organs. Following the 6 h systemic circulation, mice were sacrificed, and major organs (liver, spleen, heart, kidney, lung, brain, and intestine) were harvested for optical imaging. Representative images show the distribution of DiR-labeled UCB-NK and UC-MSC-Exos within the orthotopic A549 lung cancer model compared to PBS and healthy controls. The (P/S) represents pseudocolor scale which represents radiant efficiency. (**b**) Regional quantification of Exos accumulation. Fluorescence intensities (photons/sec/mm^2^) were quantified for each organ. Both NK-Exos and MSC-Exos exhibited primary sequestration in the liver and spleen, with significantly higher signals compared to PBS groups (## *p* < 0.01, ### *p* < 0.001, #### *p* < 0.0001). Notably, NK-Exos showed a quantifiable signal in the lung tissue of the orthotopic model, whereas MSC-Exos signal in the lungs was minimal. Proportional distribution analysis of (**c**) MSC-Exos and (**d**) NK-Exos. Pie charts illustrate the percentage of total systemic fluorescence localized within each organ after background subtraction. (**a**) Exo distribution in organs; (**b**) Quantification analysis of luminescence signals; (**c**) MSC-Exos distribution; (**d**) NK-Exos distribution.

To precisely quantify the accumulation of Exos across different tissues, major organs were harvested 6 h post-injection for high-resolution ex vivo imaging and regional analysis. Consistent with the in vivo observations, the ex vivo fluorescence imaging revealed that both UCB-NK-Exos and UCB-MSC-Exos primarily localized to the liver and spleen (Figure 4a). Quantitative analysis of the radiant efficiency showed that both Exo-treated groups (G3 and G4) exhibited a significant increase in fluorescence intensity within the liver and spleen compared to the PBS control group (##: *p* < 0.01; ###: *p* < 0.001; ####: *p* < 0.0001) (Figure 4b).

To further evaluate the organ-specific tropism of the administered vesicles, we analyzed the relative distribution percentage of DiR-labeled Exos across harvested tissues 6 h post-injection. The percentage for each organ was calculated by normalizing the background-subtracted fluorescence signal (individual organ efficiency minus the corresponding PBS control) against the total systemic fluorescence of all analyzed organs. The liver remained the primary reservoir for both vesicle types, with MSC-Exos and NK-Exos exhibiting hepatic accumulation proportions of 87.88% and 86.67%, respectively (Figure 4c,d).

The spleen represented the second-highest accumulation site, accounting for 10.10% in the MSC-Exos group and 8.57% in the NK-Exos group. Minor fluorescence signals were also detected in the intestine, representing 2.02% (MSC-Exos) and 4% (NK-Exos) of the total systemic signal. Notably, NK-Exos maintained a distinct and quantifiable presence in the lung (2.86%) and in other minor organs, with a combined signal of 0.95% across the heart, kidney, and brain (Figure 4d). In contrast, MSC-Exos were largely untraceable in these non-target tissues beyond the primary clearance organs (Figure 4c).

### 3.5. Superior Lung-Homing and Intra-Pulmonary Asymmetry of NK-Exos (Figure 5)

**Figure 5:**
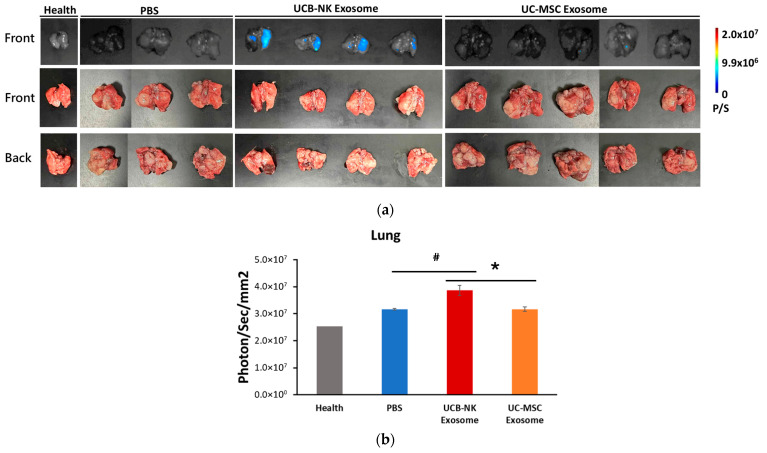
Enhanced pulmonary accumulation of NK-Exos in the orthotopic A549 lung cancer model. (**a**) Representative ex vivo fluorescence images of harvested lungs. To examine the fine-scale distribution of Exos within the pulmonary microenvironment at the 6 h endpoint, lungs were harvested and imaged from both front and back perspectives. The orthotopic A549 injection model groups (PBS, UCB-NK-Exos, and UC-MSC-Exos) are compared with healthy controls. The pseudocolor scale indicates radiant efficiency (P/S). (**b**) Regional quantification of fluorescence intensity in the lungs. The radiant efficiency (photons/sec/mm^2^) within the lung region was quantified for each experimental group. UCB-NK-Exos exhibited a significantly higher accumulation in the lungs compared to the PBS control group (# *p* < 0.05) and the UC-MSC Exos group (* *p* < 0.05). Data are presented as mean ± SEM. (**a**) Exosome dispersion in lungs at endpoint (6 h); (**b**) Analyzing Exos dispersion in lungs at endpoint (6 h).

To further investigate the pulmonary-targeting potential of NK-Exos, we performed a detailed ex vivo analysis of the lungs, specifically comparing the accumulation between NK-Exos and MSC-Exos, as well as the distribution between the tumor-bearing and healthy lung lobes. High-resolution ex vivo imaging of the harvested lungs demonstrated that UCB-NK-Exos (G3) achieved a significantly higher fluorescence intensity compared to UCB-MSC-Exos (G4) (Figure 5a). In addition, the signal of the right lung of the UCB-NK-Exos (G3) exhibited significantly higher radiant efficiency compared to the tumor-bearing left lung (where A549-luc cells were orthotopically implanted). Quantitative analysis confirmed that NK-Exos exhibited superior lung-homing capabilities, with a markedly increased radiant efficiency over the MSC-derived group (Figure 5b, *p* < 0.05, denoted by # or *).

### 3.6. Protein Cargo of NK-Exos Reveals Homing and Signaling Potential (Figure 6)

**Figure 6:**
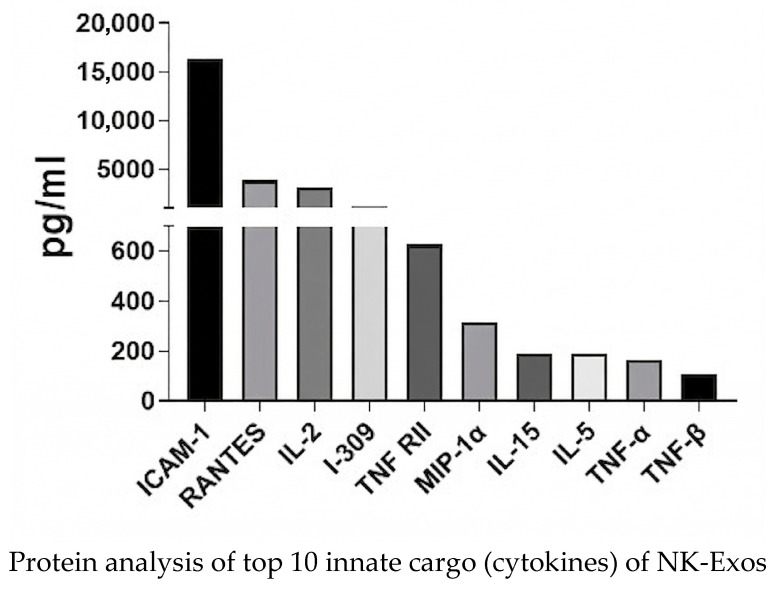
Top 10 cytokines within NK-Exos cargo via protein array analysis. The expression of the top 10 different cytokines in UCB-NK-Exos lysates was evaluated using a protein array.

To investigate the molecular mechanisms underlying the observed pulmonary tropism and immune-modulatory potential of NK-Exos, we analyzed their protein composition using a high-density antibody array. The profiling revealed the top 10 enrichment of cytokines inside of NK-Exos (Figure 6). Notably, high expression levels of ICAM-1 (CD54), RANTES (CCL5), and MIP-1a (CCL3) were detected, which involved leukocyte trafficking and cellular adhesion. Beyond trafficking molecules, NK-Exos were found to carry a distinct set of effector cytokines, including IL-15, I-309 (CCL1), and TNF RII, and TNF-α and TNF-β were also identified.

## 4. Discussion

### 4.1. Characterization and Labeling Fidelity of Therapeutic Exos

Previous studies presented that Exos exhibit superior biocompatibility, safety, and drug-loading capacity compared with synthetic nanoparticles. In addition, compared with liposomes, exosomes display enhanced targeting capability [16]. In this study, the isolated UCB-NK-Exos and UCB-MSC-Exos exhibited mean diameters of 109 nm and 114 nm, respectively (Figure 1a,b), aligning with the established size range for Exos of 30–150 nm [6]. The round-shaped morphology observed under scanning electron microscope further confirmed the structural integrity of the vesicles post-ultracentrifugation (Figure 1c). In addition, quantitative flow cytometric analysis revealed a heterogeneous distribution of the classical tetraspanins CD9, CD63, and CD81 (Figure 1d). The robust expression of CD63 and CD81 is consistent with the endosomal biogenesis pathway typical of exosomes [17]. Notably, the retention of CD56 on NK-Exos correlated to the inheritance of parent-cell immunological features, which could be analyzed in subsequent research [8].

Accurate biodistribution data relies on a labeling strategy that balances high signal intensity with minimal interference. Our results emphasize that column-based purification is indispensable for removing unbound dye. We selected the near-infrared lipophilic dye DiR, and optimized the parameters to 5 µM DiR with 2 × 10^10^ Exo particles to achieve a superior signal-to-noise ratio aligning with the dosage range of previous studies (Figure 2) [18,19].

### 4.2. Advantages of the Orthotopic A549 Model

The use of an orthotopic model instead of a subcutaneous xenograft provided a more clinically relevant platform for this study. Orthotopic tumor models preserves organ-specific vascularization, lymphatic drainage, and local microenvironmental interactions, which are crucial for studying the true biodistribution of biological carriers [17]. In addition, while few studies use the intrathoracic injection lung cancer model, more researchers utilize tail-vein injection models to mimic lung metastases [20], which often fail to replicate the complex biomechanics of a localized solid tumor. By employing a site-specific orthotopic lung cancer model via intrathoracic injection, we established a more accurate site-specific primary tumor animal platform compared to disseminated tail-vein metastatic models. Our successful establishment of the A549-luc model via intrathoracic injection allowed for real-time monitoring of tumor burden and precise localization of the Exos signal, confirming that the lung-homing of NK-Exos is a reproducible and target-specific event in a disease-state model (Figure 3).

In addition, this study was designed as an exploratory biodistribution investigation and was conducted in accordance with the 3Rs principle to minimize animal use while maintaining scientific validity. Therefore, the number of animals used in each experimental group was kept to the minimum required to permit an initial assessment of biodistribution patterns. The sample sizes were determined with reference to previously published studies employing comparable experimental designs [21].

### 4.3. NK-Exos as Potential Lung-Targeting Carriers

Our data showed that major Exos accumulated in the liver and spleen at 6 h post-injection, were consistent with previous findings (preferentially taken up by the liver and spleen with an approximate half-life of 10–30 min) [16] (Figure 4). The proportional distribution analysis (Figure 4c,d) highlights that both vesicle types are predominantly sequestered by the liver and spleen, which is consistent with the MPS-mediated clearance typical of intravenously administered exosomes [22]. Although hepatic accumulation remains the primary systemic fate, the quantifiable presence of NK-Exos in lung tissue (1.9%), compared to the negligible signals from MSC-Exos, suggests that the specific protein cargo inherited from NK cells, such as ICAM-1 and RANTES, facilitates an active organ-tropism that partially bypasses total hepatic entrapment [8]. Furthermore, the minimal accumulation in the heart, kidneys, and brain for both groups underscores a superior safety profile compared to many synthetic nanoparticle platforms, which often show significant off-target organ deposition [22]. This elevated lung-to-liver distribution ratio reinforces the potential of NK-Exos as a more specialized and efficient vehicle for targeted delivery in orthotopic lung cancer models.

Furthermore, NK-Exos have a higher affinity for lung tissue than MSC-Exos in this orthotopic model (Figure 5). This aligns with recent studies suggesting that the protein composition of NK-Exos, such as LFA-1 and various integrins, may facilitate adhesion to the pulmonary endothelium or immune cell recruitment [23]. This observation aligns with the unmet need for lung-specific delivery vehicles that can bypass rapid systemic clearance [24]. While MSC-Exos are widely recognized for their safety and low immunogenicity, their localization is often driven by passive entrapment in the pulmonary capillary bed [25].

In contrast, NK-Exos carry distinct surface markers derived from their parent cells, such as CD56 and activating receptors like NKG2D and NKp46 [26,27]. These molecules may facilitate active interactions with the pulmonary endothelium or specific components of the lung TME, leading to more robust and prolonged retention compared to the predominantly passive distribution of MSC-Exos. This lung-tropism is a critical advantage for developing NK-Exos as drug carriers to treat primary lung tumors.

### 4.4. Mechanisms Behind Differential Intra-Pulmonary Distribution

Interestingly, our MILab analysis revealed an asymmetric distribution within the lungs, with the healthy right lung exhibiting higher radiant efficiency than the tumor-bearing left lung (Figure 5). This counterintuitive finding can be explained by the physical barriers inherent in solid tumors. The elevated IFP within the tumor mass acts as a significant physiological barrier, creating an outward force that impedes the extravasation and penetration of nanoparticles, including Exos from the vasculature into the tumor core [28]. Furthermore, the presence of the orthotopic tumor in the left lung may lead to vascular compression and impaired perfusion, thereby redistributing systemic blood flow and, consequently, directing circulating Exos toward the healthy right lung [3].

### 4.5. Molecular Basis of NK-Exos Lung-Tropism

To further elucidate the molecular mechanisms driving the superior lung-homing of NK-Exos, we performed a protein array analysis. Our findings revealed that NK-Exos are enriched with key adhesion molecules and chemokines, most notably ICAM-1, RANTES (CCL5), and MIP-1α (Figure 6). The presence of ICAM-1 (CD54) on NK-Exos is particularly significant, which facilitates the docking and retention of vesicles within the dense pulmonary capillary network through homophilic or heterophilic interactions with endothelial receptors [29]. Furthermore, the cargo of pro-inflammatory chemokines like RANTES and MIP-1α supports an active lung-tropism model. These signals are known to interact with CCR1 and CCR5 receptors, highly expressed in lung tissues, guiding the vesicles toward the pulmonary microenvironment [8]. Additionally, the detection of IL-15 and TNF-α underscores that these vesicles are not merely passive carriers but functional mediators capable of sustaining immune activation. For instance, EV-associated IL-15 has been shown to enhance the persistence and activation of immune effectors within the tumor microenvironment [30]. Together, this specific protein profile provides a mechanistic explanation for the significantly higher radiant efficiency observed in the lungs of NK-Exo-treated mice, reinforcing the potential of NK-Exos as a highly targeted pulmonary drug delivery platform.

The diverse biological functions of Exos have driven a growing number of clinical trials involving Exos in recent years, indicating that Exos research is rapidly progressing from the laboratory to clinical application [16]. Owing to their nanoscale size, Exos are capable of crossing the blood–brain barrier, making them promising therapeutic carriers for neurological diseases [31]. Moreover, Exos can be surface-engineered, such as by conjugation with target-specific antibodies, to enhance their homing efficiency and tissue specificity [16].

### 4.6. Future Perspectives and Limitations: NK-Exos as Intrinsic Bio-Shuttles

Currently, the vast majority of clinical trials investigating Exos as drug delivery vehicles remain in Phase I or Phase II stages. The diverse therapeutic strategies include the direct use of unmodified plant- or stem cell-derived exosomes to deliver endogenous bioactive cargo, the application of dendritic cell-derived Exos (co-incubated with tumor antigens) as cancer vaccines, and the use of advanced genetic engineering approaches to customize mesenchymal stem cell-derived exosomes for the precise delivery of gene-editing therapeutics [32].

However, several limitations of the present study warrant consideration. First, while we observed significant pulmonary retention, the majority of the administered dose was still sequestered by the liver, highlighting the persistent challenge of the mononuclear phagocyte system barrier. However, current clinical trials have developed strategies for improving active targeting efficacy, including the surface engineering of Exos with specific ligands or antibodies [32,33]. Topical administration of Exos may also improve the efficiency of endogenous targeting compared to the systemic administration [32]. Second, our investigation focused primarily on biodistribution; future studies are required to evaluate the long-term pharmacokinetics and the therapeutic efficacy of drug-loaded NK-Exos in diverse tumor microenvironments. Despite these limitations, our work provides a critical foundation for utilizing NK-Exos as a scalable, lung-tropic delivery system for advanced respiratory malignancies.

## 5. Conclusions

The findings of this research highlight a critical challenge in drug delivery: homing to the organ does not guarantee penetration into the tumor mass. While NK-Exos are effective at reaching the target organ (the lung), overcoming the high-pressure TME remains essential for achieving deep intratumoral penetration. Future strategies may need to combine NK-Exos with IFP-reducing agents or vascular normalization therapies. The development of efficient drug delivery systems for lung cancer remains a significant challenge due to complex TME and physiological barriers. Our results demonstrate that NK-Exos possess superior lung-homing capabilities, highlighting their potential as a next-generation therapeutic carrier. Future studies should focus on combining NK-Exos with TME-modulating agents to reduce IFP, thereby enhancing the penetration of these “bio-missiles” into the primary tumor and potential metastatic nodules.

## Data Availability

The datasets generated and/or analyzed during the current study are available from the corresponding author on reasonable request.

## Supplementary Materials

The following supporting information can be downloaded at: https://www.mdpi.com/article/10.3390/life16040654/s1, Figure S1: Optimization of DiR dye concentration for exosome labeling. To determine the optimal labeling efficiency and minimize background interference, various concentrations of DiR dye were incubated with a fixed number of UCB-derived MSC-Exos. Fluorescence imaging was performed using the MILabs optical imaging system to evaluate the signal-to-noise ratio across different conditions. Experimental Groups: The test included negative controls (PBS only, 50 μM DiR dye only, 100 μM DiR dye only, and unlabeled MSC-Exos only) alongside experimental groups where 2 × 10^10^ MSC-Exos were incubated with 5 μM, 10 μM, or 50 μM of DiR dye. Imaging Parameters: Exposure time: 60,000 ms as indicated, Iris setting: f/4.0, Excitation/Emission: 710 nm/775 nm, Binning: 2 × 2. The pseudocolor intensity scale represents radiant efficiency, optimized for the detection of lipid-bound NIR dyes.

## Abbreviations

Abbreviation	Full Term
NSCLC	Non-small-cell lung cancer
UC	Ultracentrifugation
UCB	Umbilical cord blood
NK	Natural killer (cells)
MSC	Mesenchymal stem cell
Exo	Exosome
EVs	Extracellular vesicles
DiR	1,1′-dioctadecyl-3,3,3′,3′-tetramethylindotricarbocyanine iodide
HPL	Human platelet lysate
CF10	CellStack-10
GTP	Good Tissue Practice

## References

[1] Siegel R.L., Miller K.D., Wagle N.S., Jemal A. (2023). Cancer statistics, 2023. CA A Cancer J. Clin..

[2] Lorscheider M., Gaudin A., Nakhlé J., Veiman K.-L., Richard J., Chassaing C. (2021). Challenges and opportunities in the delivery of cancer therapeutics: Update on recent progress. Ther. Deliv..

[3] Heldin C.H., Rubin K., Pietras K., Östman A. (2004). High interstitial fluid pressure—An obstacle in cancer therapy. Nat. Rev. Cancer.

[4] Parakh S., Leong T.L., Best S.A., Poh A.R. (2023). Overcoming drug relapse and therapy resistance in NSCLC. Front. Oncol..

[5] Shoae-Hassani A., Hamidieh A.A., Behfar M., Mohseni R., Mortazavi-Tabatabaei S.A., Asgharzadeh S. (2017). NK cell–derived exosomes from NK cells previously exposed to neuroblastoma cells augment the antitumor activity of cytokine-activated NK cells. J. Immunother..

[6] Théry C., Witwer K.W., Aikawa E., Alcaraz M.J., Anderson J.D., Andriantsitohaina R., Antoniou A., Arab T., Atkin-Smith G.K., Jovanovic-Talisman T. (2018). Minimal information for studies of extracellular vesicles 2018 (MISEV2018): A position statement of the International Society for Extracellular Vesicles and update of the MISEV2014 guidelines. J. Extracell. Vesicles.

[7] Elsharkasy O.M., Nordin J.Z., Hagey D.W., de Jong O.G., Schiffelers R.M., Andaloussi S.E., Vader P. (2020). Extracellular vesicles as drug delivery systems: Why and how?. Adv. Drug Deliv. Rev..

[8] Federici C., Shahaj E., Cecchetti S., Camerini S., Casella M., Iessi E., Camisaschi C., Paolino G., Calvieri S., Ferro S. (2020). Natural-killer-derived extracellular vesicles: Immune sensors and interactors. Front. Immunol..

[9] Zhu L., Kalimuthu S., Gangadaran P., Oh J.M., Lee H.W., Baek S.H., Jeong S.Y., Lee S.W., Lee J., Ahn B.C. (2017). Exosomes derived from natural killer cells exert therapeutic effect in melanoma. Theranostics.

[10] Bibby M.C. (2004). Orthotopic models of cancer for preclinical drug evaluation: Advantages and disadvantages. Eur. J. Cancer.

[11] Lee O.K., Kuo T.K., Chen W.M., Lee K.D., Hsieh S.L., Chen T.H. (2004). Isolation of multipotent mesenchymal stem cells from umbilical cord blood. Blood.

[12] Spanholtz J., Tordoir M., Eissens D., Preijers F., van der Meer A., Joosten I., Schaap N., de Witte T.M., Dolstra H. (2010). High log-scale expansion of functional human natural killer cells from umbilical cord blood CD34-positive cells for adoptive cancer immunotherapy. PLoS ONE.

[13] Ni F., Zhu Q., Li H., Liu F., Chen H. (2024). Efficient preparation of high-purity and intact mesenchymal stem cell–derived extracellular vesicles. Anal. Bioanal. Chem..

[14] Heyman E., Meeremans M., Devriendt B., Olenic M., Chiers K., De Schauwer C. (2022). Validation of a color deconvolution method to quantify MSC tri-lineage differentiation across species. Front. Vet. Sci..

[15] Chen X., Su Y., Fingleton B., Acuff H., Matrisian L.M., Zent R., Pozzi A. (2005). An orthotopic model of lung cancer to analyze primary and metastatic NSCLC growth in integrin α1-null mice. Clin. Exp. Metastasis.

[16] Zhao S., Di Y., Fan H., Xu C., Li H., Wang Y., Wang W., Li C., Wang J. (2024). Targeted delivery of extracellular vesicles: The mechanisms, techniques and therapeutic applications. Mol. Biomed..

[17] Kalluri R., LeBleu V.S. (2020). The biology, function, and biomedical applications of exosomes. Science.

[18] Wiklander O.P., Nordin J.Z., O’Loughlin A., Gustafsson Y., Corso G., Mäger I., Vader P., Lee Y., Sork H., Seow Y. (2015). Extracellular vesicle in vivo biodistribution is determined by cell source, route of administration and targeting. J. Extracell. Vesicles.

[19] Mirzaaghasi A., Han Y., Ahn S.H., Choi C., Park J.H. (2021). Biodistribution and pharmacokinectics of liposomes and exosomes in a mouse model of sepsis. Pharmaceutics.

[20] Kim M.S., Haney M.J., Zhao Y., Mahajan V., Deygen I., Klyachko N.L., Piroyan A., Sokolsky M., Okolie O., Batrakova E.V. (2016). Development of exosome-encapsulated paclitaxel to overcome MDR in cancer cells. Nanomed. Nanotechnol. Biol. Med..

[21] Rebelo M., Tang C., Coelho A.R., Labão-Almeida C., Schneider M.M., Tatalick L., Ruivo P., de Miranda M.P., Gomes A., Carvalho T. (2023). De novo human angiotensin-converting enzyme 2 decoy NL-CVX1 protects mice from severe disease after severe acute respiratory syndrome coronavirus 2 infection. J. Infect. Dis..

[22] Mitchell M.J., Billingsley M.M., Haley R.M., Wechsler M.E., Peppas N.A., Langer R. (2021). Engineering precision nanoparticles for drug delivery. Nat. Rev. Drug Discov..

[23] Zhu L., Ahn B.C. (2024). Natural Killer Cell-Derived Exosome Mimetics as Natural Nanocarriers for In Vitro Delivery of Chemotherapeutics to Thyroid Cancer Cells. Exp. Oncol..

[24] Carnino J.M., Hao Kwok Z., Jin Y. (2021). Extracellular vesicles: A novel opportunity for precision medicine in respiratory diseases. Front. Med..

[25] Grange C., Tapparo M., Bruno S., Chatterjee D., Quesenberry P.J., Tetta C., Camussi G. (2014). Biodistribution of mesenchymal stem cell-derived extracellular vesicles in a model of acute kidney injury monitored by optical imaging. Int. J. Mol. Med..

[26] Lugini L., Cecchetti S., Huber V., Luciani F., Macchia G., Spadaro F., Abalsamo L., Colone M., Molinari A., Fais S. (2012). Immune surveillance properties of human NK cell-derived exosomes. J. Immunol..

[27] Di Pace A.L., Tumino N., Besi F., Alicata C., Conti L.A., Munari E., Maggi E., Vacca P., Moretta L. (2020). Characterization of human NK cell-derived exosomes: Role of DNAM1 receptor in exosome-mediated cytotoxicity against tumor. Cancers.

[28] Jain R.K., Stylianopoulos T. (2010). Delivering nanomedicine to solid tumors. Nat. Rev. Clin. Oncol..

[29] Segura E., Nicco C., Lombard B., Véron P., Raposo G., Batteux F., Amigorena S., Théry C. (2005). ICAM-1 on exosomes from mature dendritic cells is critical for efficient naive T-cell priming. Blood.

[30] Patidar M., Yadav N., Dalai S.K. (2016). Interleukin 15: A key cytokine for immunotherapy. Cytokine Growth Factor Rev..

[31] Rehman F.U., Liu Y., Zheng M., Shi B. (2023). Exosomes based strategies for brain drug delivery. Biomaterials.

[32] Liu J.J., Liu D., To S.K., Wong A.S. (2025). Exosomes in cancer nanomedicine: Biotechnological advancements and innovations. Mol. Cancer.

[33] Klyachko N.L., Arzt C.J., Li S.M., Gololobova O.A., Batrakova E.V. (2020). Extracellular vesicle-based therapeutics: Preclinical and clinical investigations. Pharmaceutics.

