# Supplementary figures and images for: Distinct Biodistribution of Natural Killer Cell-Derived Exosomes in an Orthotopic A549 Lung Cancer Mouse Model: Implications for Potent Targeted Drug Delivery
Source: Life (Basel). 2026 Apr 13;16(4):654. doi: 10.3390/life16040654 (PMC13118213; doi:10.3390/life16040654)

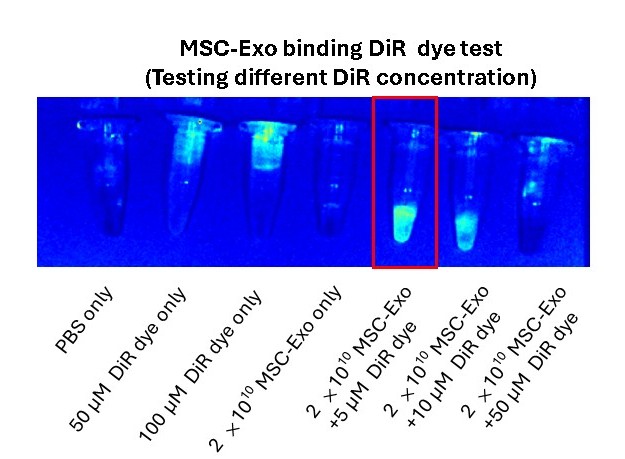

Supplement: Supplementary file 1 [file life-16-00654-s001.zip › life-4206380-supplementary.jpg]
